# Effect of Concurrent Gonadotropin-Releasing Hormone Agonist Treatment on Dose and Side Effects of Gender-Affirming Hormone Therapy in Adolescent Transgender Patients

**DOI:** 10.1089/trgh.2018.0061

**Published:** 2019-10-29

**Authors:** Rachel K. Jensen, Jennifer K. Jensen, Lisa K. Simons, Diane Chen, Ilina Rosoklija, Courtney A. Finlayson

**Affiliations:** ^1^ Northwestern University Feinberg School of Medicine, Ann & Robert H. Lurie Children's Hospital of Chicago, Chicago, Illinois.; ^2^ Division of Adolescent Medicine, Ann & Robert H. Lurie Children's Hospital of Chicago, Chicago, Illinois.; ^3^ Division of Psychiatry, Ann & Robert H. Lurie Children's Hospital of Chicago, Chicago, Illinois.; ^4^ Division of Urology, Ann & Robert H. Lurie Children's Hospital of Chicago, Chicago, Illinois.; ^5^ Division of Endocrinology, Ann & Robert H. Lurie Children's Hospital of Chicago, Chicago, Illinois.

**Keywords:** gender-affirming hormone dose, gender-affirming hormone side effect, puberty blocker, transgender youth

## Abstract

This retrospective chart review aims to address gaps in the literature regarding the efficacy and interaction of gonadotropin-releasing hormone agonists (GnRHa) and gender-affirming hormone therapies in medical transition regimens in transgender adolescents. We abstracted and reviewed data from 83 patients at our pediatric gender clinic, and found that patients who initiated treatment with GnRHa before gender-affirming hormones (estrogen, testosterone) required lower doses of those hormones than those who did not use GnRHa. The results of this preliminary research provide a foundation for future long-term prospective studies aimed to better understand these relationships.

## Background/Literature Review

Current clinical guidelines recommend the use of puberty suppressing hormones (gonadotropin-releasing hormone agonists [GnRHa]) in peri-pubertal patients diagnosed with gender dysphoria.^1,2^ GnRHa block the release of gonadotropins, inhibiting the production of endogenous sex hormones responsible for pubertal development.^3^ Pubertal suppression via GnRHa is reversible, with commencement of normal puberty upon cessation of their use.^4^ When initiated at onset of puberty in transgender youth, GnRHa can prevent irreversible physiologic changes known to exacerbate gender dysphoria and complicate future transition to the desired gender.^5^ Additionally, GnRHa administration has been associated with improved behavioral and psychological functioning and greater satisfaction with physical outcomes of gender-affirming hormones (testosterone, estrogen).^6–8^ Despite the accepted use of GnRHa among health care providers for this population, significant gaps exist in the literature regarding efficacy, side effects, and interactions of medical transition regimens for adolescent transgender patients, particularly of combined GnRHa and gender-affirming hormone regimens. Barriers to such research include lack of Food and Drug Administration approval and reliable insurance coverage for GnRHa for this indication, as well as variations in prescribed hormone regimens. Our study attempts to address some of these unknowns by isolating descriptive statistics that may provide basis for further research. Specifically, we aimed to (1) determine whether dosages of gender-affirming hormones in those taking GnRHa differ from those not taking GnRHa; and (2) identify the frequency of associated side effects in both groups. It is posited that suppression of endogenous sex hormones via GnRHa may reduce necessary doses and associated side effects of gender-affirming hormones.

## Methods

Institutional Review Board approval was granted based on the study design described below. Data from patients who began and were currently receiving gender-affirming hormone therapy at a pediatric gender clinic at a tertiary medical center before March 2016 were abstracted by a retrospective review of outpatient electronic medical records (EMR). Eighty-six patients were included in initial review, with one subject excluded due to nonbinary gender identification, as hormone doses for this individual differed from standard protocols so as to better address their specific transition goals. Data were extrapolated through January 2018 and included demographic information, health conditions and medications, GnRHa and gender-affirming hormone regimens, and reported side effects. Comorbid conditions were identified based on EMR Problem List as of the end of data collection, and recorded medications were limited to current prescriptions as of that date. Data regarding GnRHa and gender-affirming hormone dosing regimens were extrapolated from narrative and medication portions of the chart. Side effects were identified based on those recorded by care team physicians in narrative portions of EMR notes. In analyzing data regarding specific hormone regimens, we excluded two patients (one female-affirmed, one male-affirmed) who began GnRHa concurrently with gender-affirming hormones, to better distinguish between effects of GnRHa versus gender affirming hormones. Data from these subjects were included in demographic and health conditions/medication analyses. Statistical analysis included use of medians, ranges, and unpaired *T*-tests, which are reported in the tables below.

## Results

Of the 85 subjects included, 62 (73%) were male-identified, and 23 (27%) were female-identified.

The majority of subjects (72%) had subjectively reported medical comorbidities as recorded in the problem list in the EMR. The severity or chronicity of these is unknown, but common conditions included depression and anxiety, asthma, and obesity.

Prescriptions for psychiatric and nonpsychiatric medications were common, with 58/85 (68%) taking one or more medications aside from GnRHa or gender affirming medications.

Of the 17 subjects taking GnRHa, 16 were taking leuprolide, and 1 patient had a histrelin implant (replaced yearly). Among these subjects, 11 (65%) experienced noted side effects, with hot flashes, mood swings, weight gain, and fatigue being most common. During the period of data collection, 10 (59%) patients discontinued use of GnRHa, most commonly due to loss of insurance coverage. Other subjects on GnRHa experienced lapses in insurance coverage for these medications, disrupting continuity of treatment. Female-affirmed subjects had a median duration of GnRHa use of 20.6 months, with a median 14.7 month overlap in GnRHa and hormone therapy treatment (Table 1). Male-affirmed subjects had a median duration of GnRHa use of 29.3 months, with a median 23.3 month overlap in GnRHa and hormone therapy treatment (Table 2). One patient (female-affirmed) ceased GnRHa use at the time of gender-affirming hormone therapy; all other subjects on GnRHa continued to use GnRHa for at least 6 months into gender-affirming hormone treatment.

**Table 1.** Treatment characteristics in female-identified subjects

	+GnRHa	−GnRHa
*N*	6	16
Median age at initiation GnRHa	14.5 (11.4–15.7)	NA
Median duration GnRHa use (months)	20.6 (8.6–44.1)	NA
Median duration simultaneous GnRHa and hormone therapy (months)	14.7 (0.0–38.8)	NA
Median age at initiation gender-affirming hormone	14.9 (14.1–15.7)	16.7 (14.4–18.2)
Median duration follow-up after beginning gender-affirming hormones (months)	24.1 (6.4–26.9)	29.3 (7.2–53.0)
Taking spironolactone and estrogen together	0	13
Experienced side effects on estrogen	4/6 (67%)	9/16 (56%)
Most commonly reported side effects^a^	Breast tenderness (2)	Breast tenderness (7)
	Increased liver enzymes (1)	Estradiol>normal limit (2)
		Increased liver enzymes (1)
Average ending dose estradiol tablets	1.9 mg/day (SD=0.8, *N*=6)	4.6 mg/day (SD 1.5, *N*=13^b^)
Ending estradiol dose range (mg/day)	0.5–3	4–8

^a^ Based on number of times side effects noted in all charts. (Excluded from this chart are side effects experienced by only one person on estrogen.)

^b^ Three patients not included were not taking oral estradiol—were on transdermal estrogens, estradiol valerate, and no form of estrogen (discontinued).

GnRHa, gonadotropin-releasing hormone agonists.

**Table 2.** Gender-affirming hormone characteristics in male-identified subjects

	+GnRHa	−GnRHa
*N*	11	50
Median age at initiation GnRHa	13.9 (12.9–15.6)	NA
Median duration GnRHa use (months)	29.3 (13.6–51.1)	NA
Median duration simultaneous GnRHa and hormone therapy (months)	23.3 (12.3–27.3)	NA
Median age at initiation gender-affirming hormone	15.0 (13.7–16.5)	16.9 (13.4–22.1)
Median duration follow-up after beginning gender-affirming hormones (months)	26.6 (22.6–39.3)	30.4 (10.6–59.3)
Experienced side effects at any point during testosterone use	6/11 (55%)	38/50 (76%)
Most commonly reported side effects^a^	Acne (3)	Acne (26)
	Mood changes (5)	Mood changes (23)
	Increased appetite (2)	Increased appetite (12)
	Headache (1)	Headache (2)
	Hot flashes (3)	Hot flashes (1)
	Injection site rash (1)	Elevated red blood cell markers (9)
		Fatigue (3)
		Hair loss (3)
		Spotting (2)
Average ending dose subcutaneous testosterone cypionate	37.9 mg/week (SD 15.0, *n*=11)	51.7 mg/week (SD 8.4, *n*=48^b^)
Ending subcutaneous testosterone cypionate dose range (mg/week)	13.5–60	40–80

^a^ Based on number of times side effects noted in all charts. (Excluded from this chart are side effects experienced by only one person on testosterone.)

^b^ Two excluded from total because one on intramuscular (vs. subcutaneous) form, one stopped taking hormones.

In the female-affirmed population, GnRHa use beginning before estrogen was associated with a significantly lower average dose of oral estradiol at the end of the data collection period, *p*=0.0007 (Table 1). Rates of side effects of gender-affirming hormones were similar regardless of concurrent GnRHa use, with the majority in either condition reporting side effects, identified in the subjective portion of provider notes. Most commonly reported were breast tenderness, excessively elevated estradiol levels (>50 ng/dL, in accordance with Endocrine Society Guidelines), and elevated liver enzyme levels.^2^ Thirteen patients, all of whom were not taking GnRHa, were taking both spironolactone and estrogen at some point during the period of data collection.

Similarly, among male-affirmed subjects, GnRHa use correlated with lower doses of subcutaneous testosterone cypionate at the last data collection point, *p*=0.0001 (Table 2). The majority in both conditions experienced side effects, as recorded in the subjective sections of provider notes, the most common being acne, mood changes, increased appetite, and elevated red blood cell markers.

## Conclusions

GnRHa use was correlated with lower doses of gender-affirming hormones at the final point of data collection, suggesting that concurrent GnRHa may decrease doses of hormones needed to achieve desired physiologic changes. Frequency and type of side effects of gender-affirming hormones were similar regardless of whether GnRHa were prescribed, though severity of these effects was not assessed. In fact, the majority of subjects in all conditions experienced side effects of gender-affirming hormones, which appear to largely reflect changes commonly experienced in puberty, when these hormones are produced endogenously. As such, though uncomfortable, these “side effects” do not typically pose risk to the patient. More concerning are alterations in laboratory values, such as elevated liver enzymes or hematocrit, which if unaddressed can lead to organ damage.^2^ This reiterates the importance of patient monitoring in gender-affirming hormone treatment, as is currently recommended by the Endocrine Society Guidelines. Lower doses of these hormones, as appears to be reasonable with concurrent GnRHa use, may lessen the frequency and severity of these laboratory changes. Furthermore, for female-affirmed patients, those not taking GnRHa were often placed on spironolactone in addition to estrogen, which reflects the need for adjunctive therapies to achieve suppression of testosterone levels to the female range.^9^ That those on GnRHa were not placed on spironolactone may reflect diminished need for supplemental medications due to endogenous testosterone suppression by GnRHa.

Though these data support the use of GnRHa in adjunct to gender-affirming hormones, GnRHa are not without their own side effects, as noted in our findings. Side effects such as hot flashes, mood swings, and weight gain reflect the induction of hormone suppression in subjects.^2^ Though not inherently dangerous to the patient, such side effects can cause substantial discomfort and should be included in risk-benefit discussions with patients. Additionally, GnRHa have been postulated to have potential adverse effects on bone mineral density, though studies have largely been inconclusive on the true extent of this theoretical risk, and risks associated with prolonged use of GnRHa have not been examined.^2,10^ Though our chart review does not address this particular concern, we reiterate the importance of reviewing this possible risk with patients and of regular monitoring during treatment.

The aim of this retrospective chart review was to assess whether the use of GnRHa was associated with altered dosing and associated side effects of gender-affirming hormones; however, data review was notable for several additional findings that may impact care of this population.

In this cohort, high rates of subjectively reported medical and psychiatric concerns and additional medication prescriptions indicate that care of these patients often requires coordination of multiple care teams, which may influence gender-affirming treatment decisions. Additionally, many patients taking GnRHa were noted to have lapses in insurance coverage of these medications, which prevented sustained use or resulted in discontinuation entirely. Without reliable insurance coverage, patients are often unable to access GnRHa in the first place, or are subject to suboptimal treatment due to early discontinuation or inconsistent use.

## Limitations and Future Directions

Lack of standardized documentation, including specifically documented provider rationale for gender-affirming hormone prescription doses, combined with the already highly individualized nature of gender-affirming hormone regimens present a significant challenge to data collection and analysis in this study. Likewise, variation in timing of serum hormone level monitoring prevents precise correlation of serum hormones with dosage requirements. In addition, irregular patient follow-up and uncertainty of medication adherence complicate our discussion. Difficulties in acquisition and maintenance of consistent insurance coverage for GnRHa were an obstacle to our study and should be considered in the design of future research. Finally, given the retrospective nature of this study, it is impossible to isolate the effects of GnRHa therapy before versus concurrent with gender-affirming hormone therapies.

Data from this study suggest the importance of conducting large, prospective studies with more standardized methods of data collection and clearly delineated hormone protocols. Such research may better elucidate the associations suggested by this research and establish causal relationships between use of GnRHa and doses and side effects of gender-affirming hormones. Additionally, such research may provide critical evidence necessary for Food and Drug Administration approval of GnRHa for this indication, as current lack thereof poses a significant obstacle to insurance coverage of these medications for many patients.

## Abbreviations Used

EMR: electronic medical records
GnRHa: gonadotropin-releasing hormone agonists
